# Predictive value of serum inflammatory markers for histological chorioamnionitis among women with preterm premature rupture of membranes after undergoing cervical cerclage

**DOI:** 10.1016/j.clinsp.2023.100292

**Published:** 2023-10-23

**Authors:** Li Li, Xinxin Huang, Jianying Yan, Jun Zhang, Danlin Yang, Mian Pan

**Affiliations:** Department of Obstetrics, Department of Healthcare, Fujian Maternity and Child Health Hospital, College of Clinical Medicine for Obstetrics & Gynecology and Pediatrics, Fujian Medical University, Fuzhou, Fujian Province, China

**Keywords:** White blood cell, Neutrophil, Histological chorioamnionitis, Preterm premature rupture of membranes (PPROM), Cervical cerclage

## Abstract

**Purpose:**

To determine the predictive value of maternal White Blood Cells (WBC), neutrophils, and C-Reactive Protein (CRP) for diagnosing Histological Chorioamnionitis (HCA) among women with Preterm Premature Rupture of Membranes (PPROM) who underwent cervical cerclage.

**Methods:**

A retrospective cross-sectional study was conducted among women with singleton pregnancy and PPROM, who underwent cervical cerclage during 2018–2020.

**Results:**

A total of 55 eligible women were included in the final analysis, including 36 (61.02%) cases with HCA and 19 (38.98%) without HCA. Women with HCA had higher WBC count (12.31 ± 2.80) × 10^9^/L and neutrophil count (9.67 ± 2.90)×10^9^/L than those without HCA (10.35 ± 2.53) × 10^9^/L and 7.82 ± 2.82 × 10^9^/L, respectively) (both p < 0.05). The cut-off value of WBC count at 10.15×10^9^/L was found to be the most effective in identifying HCA, with an Area Under Curve (AUC) of 0.707 (95% CI: 0.56–0.86; p = 0.012), sensitivity of 86.11%, specificity of 57.90%, Positive Predictive Value (PPV) of 79.49%, Negative Predictive Value (NPV) of 68.75%, and Youden index of 0.44. The combination of WBC + neutrophil had a slightly higher (AUC = 0.711, 95% CI: 0.57–0.86; p = 0.011), specificity (68.42%), and PPV (81.25%), but lower sensitivity (72.22%), than the WBC count alone. A cut-off value of neutrophil at 7.46 × 109/L was effective in identifying HCA, with an AUC of 0.689 (95% CI: 0.53–0.84; p = 0.022).

**Discussion:**

Combination use of WBC+neutrophil was found to be the most accurate predictor of HCA among women with PPROM after surgery of cervical cerclage.

## Highlights

• Study on predictive value for HCA among women with PPROM after undergoing cervical cerclage.
• WBC and neutrophil can predict HCA among women with PPROM after surgery of cervical cerclage.
• The study provides a valuable prediction tool for early detection of HCA.

## Introduction

Cervical insufficiency is the inability of the cervix to retain the fetus, in the absence of uterine contractions or labor (painless cervical dilatation), owing to a functional or structural defect. Cervical cerclage has been widely recognized as an effective treatment for cervical insufficiency.^1^ Its aim is to provide mechanical support to the cervical canal, keep the cervix closed, and consequently prolong gestational age. The surgery itself does not increase the risk of chorioamnionitis or Preterm Premature Rupture of Membranes (PPROM) among women with cervical insufficiency.^2^

PPROM is among the most common and serious complications of pregnancy with an estimated incidence of 3% in all pregnancies and is responsible for one-third of premature deliveries.^3^^,^^4^ It refers to the Rupture of Membranes (ROM) prior to 37 weeks gestation. In recent years, increasing evidence has indicated that infections in the genitourinary tract contribute to the increased risk of PPROM. Approximately 15–25% of women with PPROM have infection prepartum, and 15–20% of women show clinical signs of infection postpartum.^5^^,^^6^ Chorioamnionitis, one of the most common types of infections in the genitourinary tract during pregnancy, has been proposed as one of the leading causes of PPROM.^7^ Among women with cervical insufficiency, the incidence of chorioamnionitis was estimated as high as 75% to 80%.^8^^,^^9^ More importantly, 42% of women with PPROM were observed with chorioamnionitis.^10^

Results from previous studies have indicated that chorioamnionitis is one of the leading causes of premature delivery among women with cervical insufficiency after cervical cerclage. Diagnosis of chorioamnionitis is very important to guide clinical treatment in pregnancies complicated by PPROM. Upon confirmation of chorioamnionitis, cerclages should be removed instantly to terminate the pregnancy. In clinics, chorioamnionitis can be divided into two subtypes: clinical chorioamnionitis and subclinical (or Histological Chorioamnionitis [HCA]). Although clinical chorioamnionitis can be characterized by maternal fever and other symptoms ‒ for example, maternal leukocytosis (> 15,000 cells/mm^3^) and maternal tachycardia (> 100 bpm) ‒ there are no obvious early symptoms for HCA. Currently, amniotic fluid inflammation biomarkers including White Blood Cells (WBC) (≥ 50 cells/mm^3^), glucose (≤ 14–15 mg/dL), IL-6 (> 2.5–2.6 ng/mL), and Matrix Metalloproteinase (MMP)-8 (> 23 ng/mL) are suggested as valuable predictors for early diagnosis of HCA.^11^, ^12^, ^13^ However, amniocentesis is not regularly recommended because of its invasive nature.^14^ Therefore, it is truly critical to identify non-invasive biomarkers for the early diagnosis of HCA in pregnant women complicated with PPROM for further improvement of clinical practices.

Changes in serum inflammatory biomarkers detected in pregnant women's peripheral blood are generally believed to appear earlier than the clinical symptoms of chorioamnionitis. Several serum inflammatory biomarkers including C-Reaction Protein (CRP), platelet count, Neutrophil-Lymphocyte Ratio (NLR), and Platelet-to-White Blood Cell ratio (PLT/WBC) have been suggested as potential diagnostic markers of either clinical or histological chorioamnionitis.^15^, ^16^, ^17^, ^18^, ^19^ However, to the best of our knowledge, no studies have discussed the predictive value of serum biomarkers for diagnosis of HCA among pregnant women with PPROM who have undergone cervical cerclage. Therefore, the current study aimed to explore the predictive value of serum biomarkers in detecting HCA among pregnant women with PPROM after undergoing cervical cerclage. The findings of this study could provide novel evidence on the application of serum biomarkers for the early detection of HCA.

## Material and methods

### Study patients and study design

The present retrospective cross-sectional study was conducted among women with singleton pregnancy and PPROM, who underwent cervical cerclage in Fujian Provincial Maternity and Children's Hospital, Fujian, China between January 1, 2018, and December 31, 2020. The study was approved by the Ethics Committee of Fujian Provincial Maternity and Children's Hospital, Fujian, China (nº 2021KRD018). All patients signed the written informed consent.

The inclusion criteria were as follows: 1) Gestational age ≤ 36 weeks and 6 days; 2) Underwent vaginal MacDonald cervical cerclage; 3) Singleton pregnancy; 4) Spontaneous PPROM after surgery of cervical cerclage; 5) Treated with antibiotics for at least 1-week after PPROM; 6) Treated with betamethasone therapy for fetal lung maturity and tocolysis after PPPROM; 7) No clinical manifestations of chorioamnionitis, such as fever, abnormal maternal heart rate, and abnormal fetal heart rate; and 8) Time interval between ROM and delivery was ≤ 3 days. A ≤ 3-day interval between ROM and delivery is assumed to be appropriate, as the relationship between the index test and the placental outcome measurement would be preserved.^20^

The exclusion criteria were as follows: 1) Multiple pregnancies; 2) Underwent multiple surgeries of cervical cerclage during this pregnancy; 3) Term birth after cervical cerclage; 4) Fetal malformation; 5) Rupture of membranes occurred during surgery of cervical cerclage; and 6) Underlying comorbidity of diabetes, hypertension, or placenta previa.

The sample size (n) was calculated by using the following formula:^21^ $n=\frac{P\left(1-P\right)z_{1-\alpha /2}^{2}}{d^{2}}$, P is the prevalence of chorioamnionitis in PPROM that was equal to 0.15‒0.25,^22^ margin of error (d) considered as 0.12 and type one error (a set at 0.05), it was calculated that 51 women would be required to observe a significant difference between groups.

### Treatment

Women with a diagnosis of PPROM after surgery of cervical cerclage were treated with antibiotics (intravenous drip cefmetazole) after hospital administration. For women at a gestational age of 26–34 weeks, corticosteroids were administered to promote fetal lung maturity, and an intravenous drip of magnesium sulfate was used to protect the fetal cerebrum. If uterine contractions occurred within 48h after hospital administration, tocolytics (ritodrine hydrochloride or Atosiban) were also used. The procedure for cerclage removal complied with the American College of Obstetricians and Gynecologists Practice guidelines.^23^

### Data collection

The following baseline characteristics were collected: 1) Demographic information: maternal age (years); 2) Obstetric data: multipara (yes vs. no), history of miscarriage in the second trimester (yes vs. no), history of miscarriage (yes vs. no), history of preterm birth (yes vs. no), history of hysteroscopy (yes vs. no), gestational ages at cerclage (weeks), gestational age at PPROM (weeks), placental abruption (yes vs. no), gestational age at delivery (weeks), cesarean section (yes vs. no), clinical chorioamnionitis (n, %), birth weight (grams), and Apgar score (1-min, 5-min, and 10-min); and 3) Laboratory testing based on maternal peripheral blood: WBC count, neutrophil count, platelet count, Neutrophil-to-Lymphocyte Ratio (NLR), Platelet-to-WBC Ratio (PWR), and CRP. Peripheral blood specimens were collected upon hospital admission for treatment of PPROM. Serological analysis was performed by the Department of Clinical Laboratory of the hospital. CRP was assessed based on immune scatter turbidity. Clinical chorioamnionitis was diagnosed when the maternal temperature was elevated to 37.8°C together with any two of the following criteria presented: uterine tenderness, malodorous vaginal discharge, maternal leukocytosis (≥ 15,000 cells/mm^3^), maternal tachycardia (≥ 100 beats/min), and fetal tachycardia (≥ 160 beats/min).^24^

### Diagnosis of HCA

Analysis of placental histology was used as the gold standard for diagnosis of HCA. After delivery, tissue samples were obtained from the placenta, umbilical cord, and placental membranes, and the samples were treated with alcohol-xylene and paraffin. Using a microtome, 3-um-thick sections were cut. These sections were stained with hematoxylin-eosin and examined under a light microscope for histological signs of neutrophil infiltration and HCA. All tissue samples were evaluated by the same pathologist. HCA was defined as the infiltration of the amnion, chorion, and parietal deciduae with maternal neutrophils.

### Statistical analysis

The study subjects were divided into two groups: those with a confirmed diagnosis of HCA, and those without HCA. Continuous variables were expressed as the mean ± Standard Deviation (SD) for data with approximate normal distribution, or median (Interquartile, IQR) for data with non-normal distribution. Numbers and percentages (%) were reported for categorical variables.

The Shapiro-Wilk or Kolmogorov-Smirnov test was used for testing the normality of data. Chi-Square test or Fisher exact probability method was used for the comparison of counting data. Independent *t-*test or *t*’-test was used to compare data of normal distribution between two groups, and rank sum test was used for non-normal distribution.

The sensitivity, specificity, Positive Predictive Value (PPV), and Negative Predictive Value (NPV) of each test were calculated with respect to HCA diagnosis. The Receiver Operator Characteristic (ROC) curve (sensitivity vs. 1-specificity) was plotted for each factor, and the Area Under the Curve (AUC) was estimated. An AUC value of 1 indicated a perfect test, a value of > 0.9 indicated high accuracy, and a value of 0.7–0.9 indicated moderate accuracy. Values < 0.7 were considered to indicate low accuracy.^25^ The Youden index was used to determine the cut-off value of WBC and neutrophils in predicting HCA. A two-sided p < 0.05 was considered to indicate statistically significant differences. All data analyses were conducted using SPSS version 24.0 (IBM Corporation, Armonk, NY, USA).

## Results

A total of 55 women with spontaneous PPROM after cervical cerclage were enrolled in the study (Table 1). Among these women, 36 (61.02%) were diagnosed with HCA. The mean (±SD) age of women with HCA and those without HCA (31.00 ± 5.40 years vs. 33.74 ± 5.21 years; p = 0.081) was similar. Gestational age at PPROM and delivery were significantly lower in the HCA than the non-HCA group (all p < 0.05). The prevalence of clinical chorioamnionitis among HCA women was 63.90% (23/36); there was no case of chorioamnionitis in the non-HCA group (p < 0.001). The median fetal birth weight was 1405.42 ± 838.31g in the HCA women group and 2245.0 ± 783.39 g in the non-HCA group (p < 0.001). Apgar scores at 1-min, 5-min, or 10-min were not significantly different between the two patient groups (all p > 0.05).

**Table 1:** Demographic and clinical characteristics (Perform *t*-test and χ^2^-test).

	Histological chorioamnionitis [χ±s/n (%)]			
	Yes (*n* = 36)	No (*n* = 19)	χ^2^/*t*	*p*-value
Maternal age, y	31.00 ± 5.40	33.74 ± 5.21	-1.831	0.081
Multipara			0.164	0.685
Yes	15 (41.70)	9 (47.37)		
No	21 (58.30)	10 (52.63)		
History of miscarriages in the second trimester			0.115	0.734
Yes	8 (22.2)	5 (26.3)		
No	28 (77.8)	14 (73.7)		
History of miscarriages			0.368	0.544
Yes	14 (38.9)	9 (47.4)		
No	22 (61.1)	10 (52.6)		
History of preterm birth			0.245	0.621
Yes	4 (11.11)	3 (15.79)		
No	32 (88.89)	16 (84.21)		
History of hysteroscopy			0.322	0.571
Yes	8 (22.22)	3 (15.79)		
No	28 (77.78)	16 (84.21)		
Gestational age at cerclage, wk	22.68 ± 3.22	22.81 ± 2.55	0.164	0.879
Gestational age at PPROM, wk	27.53 ± 4.80	31.8 ± 4.74	3.222	0.002
Gestational age at delivery, wk	28.14 ± 4.67	32.21 ± 4.43	3.536	0.001
Placental abruption			1.628	0.202
Yes	9 (25.00)	2 (10.53)		
No	27 (75.00)	17 (89.47)		
Cesarean section			3.220	0.073
Yes	7 (19.44)	8 (42.11)		
No	29 (80.56)	11 (57.89)		
Clinical chorioamnionitis			20.864	< 0.001
Yes	23 (63.90)	0 (0)		
No	13 (36.10)	19 (100.00)		
Birth weight, g	1405.42 ± 838.31	2245.00 ± 783.39	3.610	< 0.001
Apgar score (1 min)	8.68 ± 2.17	9.72 ± 0.58	2.289	0.055
Apgar score (5 min)	9.21 ± 2.17	9.94 ± 0.24	1.652	0.160
Apgar score (10 min)	9.50 ± 1.32	10.00 ± 0.00	1.857	0.117

**Fig. 1:**
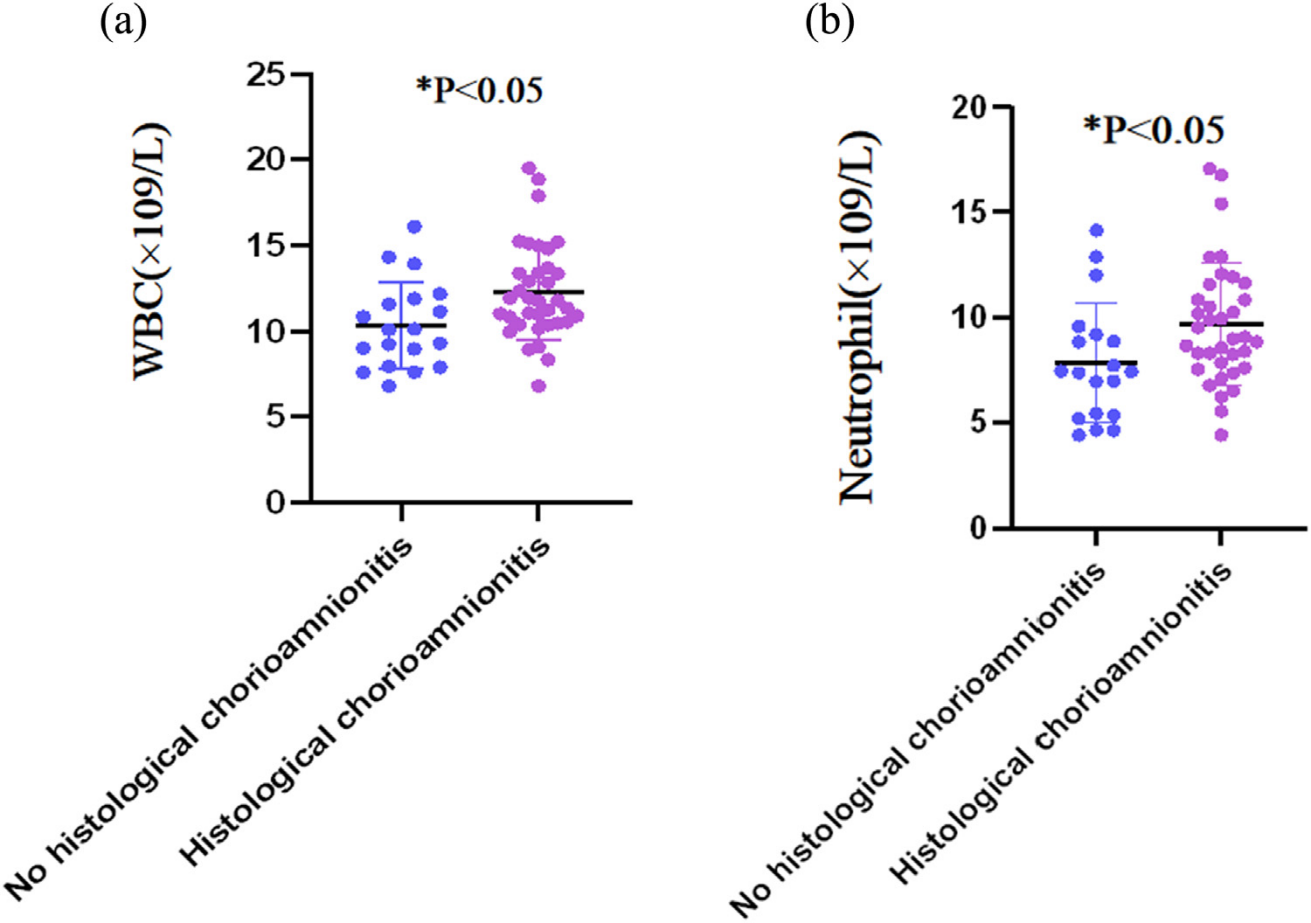
Distributions of serum inflammatory cells: (1A), white blood cell; (1B), neutrophil.

Table 2 summarizes the comparisons of serum biomarkers between two patient groups. Women with HCA had significantly higher WBC count (12.31 ± 2.80) × 10^9^/L and neutrophil count (9.67 ± 2.90) × 10^9^/L than those without HCA (10.35 ± 2.53 × 10^9^/L and 7.82 ± 2.82 × 10^9^/L, respectively) (both p < 0.05). The serum CRP level was similar between the HCA and non-HCA groups (7.95 mg/dL [IQR: 0.73–25.44] and 6.50 mg/dL [IQR: 2.08–11.85, respectively; p = 0.430). No statistically significant intergroup differences were observed for platelet count, NLR, and PWR (all p > 0.05). Fig. 1 further illustrates the distributions of WBC and neutrophil counts in both groups.

**Table 2:** Comparisons of maternal serum inflammatory biomarkers (Perform *t*-test and *z*-test).

	Histological chorioamnionitis [χ±s/P_50_(P_25_,P_75_)]		*t*/z	*p*-value
	Yes (*n* = 36)	No (*n* = 19)		
White blood cell (×10^9^/L)	12.31 ± 2.80	10.35 ± 2.53	2.558	0.013
Neutrophil (×10^9^/L)	9.67 ± 2.90	7.82 ± 2.82	2.261	0.028
Platelet (×10^9^/L)	208.00 ± 37.56	222.94 ± 42.20	1.295	0.201
Neutrophil-to-lymphocyte ratio	7.05 ± 4.52	5.43 ± 3.80	1.332	0.189
Platelet-to-white blood cell ratio	21.14 ± 5.88	18.87 ± 5.03	1.502	0.139
C-reactive protein^a^ (mg/dL) (IQR)	7.95 (0.73, 25.44)	6.50 (2.08, 11.85)	0.788	0.430

IQR, Interquartile Range.

a C-reactive protein normality test result: p < 0.001 for Kolmogorov-Smirnov and p < 0.001 for Shapiro-Wilk.

**Fig. 2:**
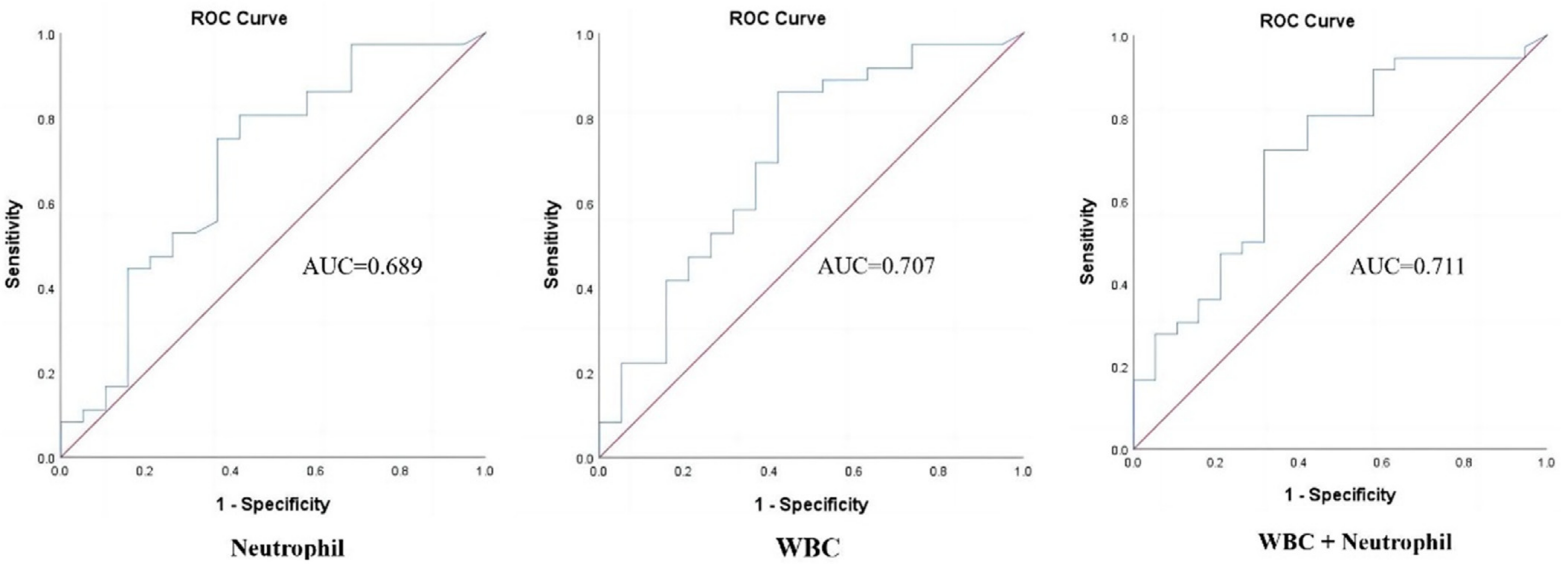
The Receiver-Operating Characteristic (ROC) curves for serum White Blood Cell (WBC), neutrophil, and combined inflammatory factors (WBC+neutrophil) to predict histological chorioamnionitis.

The accuracy of each significant parameter to predict HCA was evaluated by the ROC curve (Fig. 2). The WBC count cut-off value of 10.15×10^9^/L was found to be the most effective in identifying HCA, with an AUC of 0.707 (95% CI: 0.56–0.86; p = 0.012), sensitivity of 86.11%, specificity of 57.90%, PPV of 79.49%, NPV of 68.75%, and Youden index of 0.44 (Table 3). The combination of WBC+neutrophils had a slightly higher (AUC = 0.711, 95% CI: 0.57–0.86, p = 0.011), specificity (68.42%), and PPV (81.25%), but lower sensitivity (72.22%) than WBC count alone. The cut-off value of neutrophils at 7.46×10^9^/L was found to be the most effective in identifying HCA, with an AUC of 0.689 (95% CI: 0.53–0.84; p = 0.022).

**Table 3:** Diagnostic indices of serum White Blood Cell (WBC), neutrophil and combined inflammatory factors (WBC+neutrophil) in study subject (Perform Fisher's test).

	AUC	95% CI		p-value	Sensitivity	Specificity	PPV	NPV	Youden index
White blood cell^a^	0.707	0.56	0.86	0.012	86.11	57.90	79.49	68.75	0.44
Neutrophil^a^	0.689	0.53	0.84	0.022	80.56	57.90	78.38	61.11	0.39
White blood cell+neutrophil	0.711	0.57	0.86	0.011	72.22	68.42	81.25	56.52	0.41

AUC, Area Under Curve; SE, Standard Error; PPV, Positive Predictive; NPV, Negative Predictive Value.

a Cutoff value was 10.15×10^9^/L for white blood cell; 7.46×10^9^/L for neutrohil. Value.

## Discussion

Among women with PPROM who underwent cervical cerclage, no consensus has been reached for early prediction of HCA. In this retrospective study, the authors found that both maternal serum WBC count and a combination of WBC+neutrophils had a moderate prediction ability for HCA diagnosis. This finding might provide novel evidence on the application of serum biomarkers for the early detection of HCA in clinical practices.

The serum WBC count has been widely recommended as a predictor for the diagnosis of chorioamnionitis. Musilova et al. assessed the maternal WBC count of 479 women complicated by PPROM.^26^ The results showed that women with both Microbial Invasion of the Amniotic Cavity (MIAC) and Intra-Amniotic Inflammation (IAI) (median: 14.0 × 10^9^/L) had higher WBC count than those with either IAI alone (12.1 × 10^9^/L) or MIAC alone (12.1×10^9^/L) and those without MIAC and IAI (median: 11.8 × 10^9^/L) (all p < 0.05). The estimated WBC count in the current study was 12.31 ± 2.80 × 10^9^/L, which is comparable to results reported by Musilova et al.^26^ Furthermore, the current study reported an AUC value of 0.707, a sensitivity of 86.11%, and specificity of 57.90%, indicating that the WBC count had a moderate ability in predicting HCA. These results are comparable to a study by Nasrin et al.^19^ (AUC = 0.6, sensitivity = 82.8%, specificity = 43.5%). However, it should be mentioned that the WBC count cut-off value in the current study was 10.145 × 10^9^/L, which overlapped with the normal reference value of WBC count. This might partly explain the high sensitivity but low specificity for the WBC count, as misdiagnosis cannot be avoided in women without HCA.

Neutrophils are usually used as a biomarker for the diagnosis of inflammation, sepsis, and infection. Previous studies have reported that maternal neutrophil count could be used as a potential predictor for the diagnosis of PPROM and inflammation. Lyubomirskaya et al. reported that when the percentage of neutrophils is > 76%, the AUC value for predicting PPROM was 0.847.^27^ Moreover, the risk of developing PPROM at 26–34 weeks of gestation with subsequent preterm delivery increased in the presence of neutrophils > 76% (OR = 34.47, 95% CI: 9.67–122.88).^27^ Another study also reported an increased level of maternal neutrophil among women with impending preterm birth. The neutrophil count had an AUC value of 0.706 with a cut-off value of 10865, sensitivity of 49.6%, and specificity of 85.6% in predicting placental inflammatory response.^28^ By contrast, the current study reported a similar AUC value of 0.689, but higher sensitivity (80.56%) in predicting HCA among women with PPROM after undergoing cervical cerclage.

CRP is a nonspecific biomarker during the inflammation process and has been widely used to monitor various inflammatory conditions including chorioamnionitis. However, results from earlier studies regarding the use of maternal CRP to monitor chorioamnionitis are inconsistent. Jung et al. showed that maternal CRP is a potential predictor for intra-amniotic inflammation among pregnant women with cervical incompetence, and CRP shared a similar predicting ability as amniotic fluid WBC count.^29^ In another study, Nasrin et al. observed that maternal CRP at admission and before delivery had a good ability to predict chorioamnionitis.^19^ Specifically for CRP value before delivery, the predicting sensitivity was 83%, with a specificity of 73%, PPV of 73%, and NPV of 97% at a cut-off value of 16 mg/dL. However, the results of a systematic review and meta-analysis showed a rather low predicting ability for CRP in the diagnosis of chorioamnionitis.^30^ A recent meta-analysis of five studies with 252 participants to assess the performance of maternal serum CRP in predicting HCA showed that the sensitivity and specificity for CRP ≥ 20 mg/L was 59% (95% CI: 48–69) and 83% (95% CI: 74–89), respectively.^20^ Similar conclusions were also reported by another meta-analysis that analyzed data from 13 studies.^30^ The present study did not find statistically significant differences in maternal serum CRP between women with and without HCA; this could be partly attributed to the small sample size.

The current study indicates that a combination use of multiple maternal serum biomarkers could enhance the ability to predict HCA. Combined WBC and neutrophil count had the highest AUC value of 0.711. Although the sensitivity has decreased slightly, the specificity and PPV were both higher than the single parameter. A study used a combination of maternal serum neutrophil cell count, lymphocyte count, and CRP to predict placenta infection, in which the estimated AUC value was 0.746, with a sensitivity of 69.1% and specificity of 70.5%.^31^ The combination use of multiple biomarkers is beneficial to overcome the limitation of using a single biomarker and increase the accuracy of prediction.

The main strength of the current study is that the authors only recruited women with an interval of ≤ 3 days between ROM post cervical cerclage and delivery. If the time interval between ROM and delivery is prolonged, the infection status at delivery might not effectively and accurately project that status at ROM. Meanwhile, selection bias could be avoided by excluding women with complications of pregnancy that might result in PPROM. However, this study also has some limitations. Retrospectively collected data from a single center were used. Furthermore, the statistical power is also limited by the small sample size. Future studies should include a larger sample size from multiple centers to validate these findings.

## Conclusion

For women diagnosed with PPROM after cervical cerclage, early detection of chorioamnionitis is of utmost importance to provide timely treatment. Results from the present study indicate that maternal serum WBC and a combination of WBC and neutrophils could be potentially used as a valuable prediction tool for early detection of HCA.

## Ethics approval and informed consent

The study was approved by the Ethics Committee of Fujian Provincial Maternity and Children's Hospital, Fujian, China (nº 2021KRD018) and conducted in accordance with the Declaration of Helsinki. All participants provided written informed consent, and the potential risks were clearly explained to them. All responses were collected and analyzed without identifiers. This study followed the STARD guidelines.

## Consent for publication

N/A.

## Data availability statement

The data that support the findings of this study are available on request from the corresponding author. The data are not publicly available due to privacy or ethical restrictions.

## Authors' contributions

Mian Pan: Conceptualization, Methodology, Project administration, Funding acquisition, Writing-review, and editing

Jianying Yan: Conceptualization, Methodology, Writing-review, and editing

Jun Zhang: Data curation, Writing-review, and editing

Danlin Yang: Data curation, Writing-review, and editing

Li Li: Formal analysis, Writing-original draft, Writing-review, and editing

Xinxin Huang: Formal analysis, Result interpretation, Writing-review, and editing

## Funding

This work was supported by the Natural Science Foundation of Fujian Province (No. 2021J01406). The funders had no role in study design, data collection and analysis, decision to publish, or preparation of the manuscript.

## Declaration of Competing Interest

The authors declare no conflicts of interest.
